# Materia Medica and Therapeutics; Including the Preparations of the Pharmacopœias of London, Edinburgh, Dublin, and the United States; with Many New Medicines

**Published:** 1847-04

**Authors:** 


### BIBLIOGRAPHICAL NOTICES.

---

Art. V.—*Materia Medica and Therapeutics; including the Preparations of the Pharmacopœias of London, Edinburgh, Dublin and of the United States: with many New Medicines.* By J. Forbes Royle, M.D., F.R.S., Late of the Medical Staff of the Bengal Army; Member of the Medical and Chirurgical Society of London: of the Medical aud Physical Society of Calcutta; and of the Royal Medical Society of Edinburgh, etc.: Professor of Materia Medica and Therapeutics, King's College, London. Edited by Joseph Carson, M.D., Professor of Materia Medica in the Philadelphia College of Pharmacy; Member of the American Philosophical Society, etc. etc.: With ninety-eight Illustrations. pp. 690. Philadelphia: Lea and Blanchard. 1847.

This is a neat, well executed volume of nearly seven hundred pages, with a full index, and presents a condensed view of Materia Medica. As a text-book for students it strikes us as being just the volume wanted in this country. Pereira is too voluminous for this class of readers, and no work now occurs to us as so well adapted to their wants as the one the title of which stands at the head of this article.

The author has adopted the natural history arrangement, in preference to the one which classifies medicines according to their operation. We do not conceive that this is the best; we should prefer the physiological arrangement, as giving at one view all the articles fitted to fulfil the same indication. But the objection which might have been raised to his work on this account the author has obviated, by giving at the end of the volume a list of all the medicines noticed in it, arranged with reference to their therapeutical effects. By referring to these tables the student may soon fix in his mind the remedies belonging to the same class.

The volume opens with definitions such as the student needs at the threshhold of his studies, and this is followed by remarks on operations of pharmacy, which will be found highly useful to the beginner. Taking up first the articles belonging to the inorganic kingdom, atmospheric air receives a brief consideration, and then follow oxygen, nitrogen, hydrogen, water, sulphur, etc. In connection with sulphur, sulphuric acid is described, and all that relates to its production and properties is presented in the narrowest practicable limits. The author has been able to carry his condensation farther, by adopting symbols and abbreviations. Thus, for sulphuric acid, he gives S′ or $SO^3 = 40$, which tells its composition and combining number; and in referring to it afterwards he uses S′ or Sul′ instead of writing out the word in full. In like manner N′ or nitric′, stands for nitric acid, and carb′ for carbonic acid, and so on. This is an excellent innovation, by which time is saved, and the size of the volume has been kept within better limits. As at once a specimen of the work, and containing what may be acceptable to our readers, we quote the article on the *iodide of iron:*

### Ferri Iodidum, L. E. (U. S.)

Iodide of Iron. *Ioduret and hydriodate of Iron.* *F.* Iodure de Fer. *G.* Eisen Iodur.

Introduced into practice by Dr. A. T. Thomson, and described in his Obs. on the Prep. and Med. Employment of the Iodurets and Hydriodate of Iron.

*Prop.* Iodide of Iron (Fe I+5 Aq.=199) is of a dark gray color, with somewhat of a metallic appearance; its taste is acrid and styptic. It is often prepared in thin cakes, of a crystalline radiated structure and light gray color when fractured. If its solution be evaporated with as little contact of air as possible, "green tabular crystals are formed." It is very deliquescent, and readily dissolved in water, as also in Alcohol. The solution, when pure, is colorless, and when diluted, is not disagreeable. Heated, it volatilizes and readily fuses; but is then easily decomposed, Iodine escaping in vapor, and Iron being left in the form of Sesquioxide. From the absorption of Oxygen, the same change takes place on exposure to the air: water is absorbed to the extent of forming a dark-coloured solution, in which some Sesquioxide of Iron with a little free Iodine is held in solution, and a Sesquioxide of Iron precipitated. It is difficult to preserve it even in solution, unless a coil of Iron wire, as suggested by Mr.

Squire, be introduced into it. Sugar also has been ascertained to have this preservative effect. Comp. Fe 14+I 63·3+Aq. 22·7 = 100.

*Prep.* L. E. Take *Iodine* ℥vj. (dry gr. 200, E.) *Iron Filings* ℥ij. (fine iron wire recently cleaned gr. 100, E.) *Aq. dest.* Oivss. (f℥vj. E.) Mix the Iodine with Oiv. of the water, add the Iron, heat them in a sand-bath (boil in a glass matrass, first gently, to avoid the expulsion of Iodine vapor, afterwards briskly till concentrated to one-sixth its volume, E.) till the solution becomes pale green, pour off the liquid, wash the residue with the remaining boiling *Aq.* Oss. and add this to the other liquid, L. Filter. Evaporate to dryness in an iron vessel, at a temperature not exceeding 212°. (Put the filtered liquid quickly into an evaporator, with 12 times its weight of Quicklime round the basin, in an apparatus where it may be shut up in a small space, not in contact with the general atmosphere. Heat the whole apparatus, till the water be evaporated, E.) Preserve the product in (small, E.) well-closed vessels (excluded from the light, L.)

The Messrs. T. and H. Smith now make a solution of Iodide of Iron in a Florence flask with ʒvj. of pure iron filings, ℥ij. ʒij. of Iodine and f℥ivss. of cold distilled water. Boil till the liquid loses its color, and filter rapidly into another clean flask, and evaporate at a boiling heat. They obtain the compound either as a crystalized hydrate, or in an amorphous anhydrous form, according to the extent of the evaporation, and enclose without the smallest delay in small well-corked bottles. Mr. Kop recommends triturating 4 parts of Iodine with 2 parts of water, in a large dish; then to add at once, 1 part of iron filings in a state of fine division, and to continue the trituration.

[U. S. *Iodine* ℥ij. *Iron Filings* ℥j. *Water* Oivss.]

Of these preparations that of E. P. is preferable. The solution, like that of all the protosalts of Iron, is of a green color. If this be quickly filtered and evaporated, and with as little access of air as possible, the salt may be obtained without much decomposition; but, as the iron is apt to pass readily to the state of Sesquioxide, it is best prepared according to the Messrs. Smith's improved formula. As all the solid preparations are liable to change, they further recommend powdering their anhydrous Iodide as soon as it is taken from the flask, and then instantly to incorporate it with twice its weight of pure refined Sugar in powder, and to make it into a mass with honey. 4 grains will contain 1 grain of the Iodide.—Keep in shallow corked bottles in a layer of some powder.

(P. J. iii. 490.) Mr. Kop's preparation, it is said, may easily be administered in the form of pills."

About a third of the volume is devoted to inorganic substances, vegetable and animal materia medica claiming the larger portion. First some notice is taken of *flowering plants*, their organs of vegetation and of reproduction, subjects of which medical students should possess some knowledge; then a very brief allusion is made to flowerless or cryptogamic plants, after which we have their classification, and a very interesting account of their proximate principles, their geography, and the mode of collecting and drying them. The dicotyledonous plants are first considered, including, among others, hellebore, aconite, columba, cocculus Indicus, and poppy with all its products—substances as variant in their therapeutical action as any belonging to medicine. This classification, although inconvenient for reference, has this advantage, that it shows the botanical relations of the articles of the materia medica. We give an extract from this division of the work:

"Büttneriaceæ. *R. Brown.*

*Theobroma Cacao*, or Cacao-tree, though not officinal, is interesting in consequence of its seeds being largely employed in diet. The tree is a native of Mexico, but extensively cultivated in the West India Islands, and remarkable for its large and oval, yellow, cucumber-like capsules, hanging from the sides of the trunk and branches. These are divided into five cells, each filled with 8 to 10 ovoid seeds, piled one upon another, and covered by a membranous and succulent aril. There are other varieties of these seeds or *nibs*, which are more or less esteemed. The kernels of the seeds yield by pressure about one half of their weight of a fatty oil, commonly called *Butter of Cacao*, at one time much lauded for its medical properties. The seeds, pounded, digested, and boiled with water, with the oil skimmed off, and sweetened with sugar and milk, afford a wholesome and agreeable beverage. The Cocao sold in the shops consists either of the roasted kernels and husks, or of the husks only, ground to powder. It is sometimes made from the cake left after expressing the oil from the beans. "Much of the cheap stuff sold under this name, is very inferior, being made with damaged nuts that have been pressed for the oil, mixed with potato-flour, mutton suet, &c." (*Cooley.*) *Flake Cocao* is Cocao ground, compressed, and flaked by machinery. CHOCOLATE, (from the Indian name *chocolat*) is made by triturating in a heated mortar the roasted seeds without the husks, 10 lbs. with an equal quantity of Sugar, and about 1½ oz. of Vanilla, and 1 ounce of Cinnamon (*Cadet*) into a paste, which is put up in various forms. "The mass of common Chocolate sold in England is prepared from the cake left after the expression of the oil, and this is frequently mixed with the roasted seeds of ground peas and maize, or potato-flour, to which a sufficient quantity of inferior brown sugar, or treacle and mutton suet is added, to make it adhere together. (*Cooley.*)

*Action. Uses.* Both cocao and chocolate form the basis of very nourishing and agreeable beverages (whence the name of Theobroma, or food for the gods) devoid of the stimulating properties of tea and coffee, but apt to disagree with some people and with many dyspeptics, in consequence of the quantity of oily matter they contain."

With another quotation we take leave of the work, recommending it cordially to the profession as not only one of the most tasteful and elegant, but as one of the most valuable that the subject has yet called forth.

"ACIDUM OXALICUM. Oxalic Acid. *Acid of Sugar.*

Oxalic acid ($C^2 O^3 + 3$ Aq.= 63 when crystalized) has obtained its name from the foregoing plant. It is said to be contained in a free state in cicer arietinum, but is probably in the state of binoxalate. It is acid and powerful enough to blanch the boots in walking through a field of the plant. Some lichens contain a very large proportion of oxalate of lime. It is now obtained in the largest quantities from the action of nitric acid on several substances of the nature of sugar and starch, including these substances themselves. Hence it has been called acid of sugar. The nitric becoming decomposed, these substances lose their hydrogen, become oxidized and converted into an acid, which is found to be composed of 2 Eq. of carbon united with 3 of oxygen. This is soluble in about its own weight of hot, and about 8 times its weight of cold water, the solution being intensely acid. It readily crystalizes in quadrangular crystals, which are colorless and transparent, elongated, six-sided, and flattened, with two or four terminal planes, being derived from an oblique rhombic prism. The crystals effloresce in a dry atmosphere, and melt in their water of crystalization, are volatilized by heat, decomposed at a higher temperature, and with the aid of sul′ into water, carb′, and carbonic oxide. Their acidity is powerful, acrid, and corrosive: hence it is a virulent poison. Numerous fatal cases have occurred from the resemblance of its crystals to to those of epsom salts. But they may readily be distinguished by their crackling noise when dissolving in water; by the intensely acid taste and reaction of the solution; by its effervescing with the alkaline carbonates, which give a white precipitate with epsom salts or sulphate of magnesia (p. 129). This is moreover distinguished by its nauseously bitter taste. The crystals of ox′ also resemble those of sulphate of zinc (p. 158). Oxalic′ is distinguished by its powerful affinity for lime, separating it even from sulphuric′. The oxalate of lime formed is insoluble in an excess of acid. A soluble oxalate will be detected by the solution of a neutral salt of lime or of oxide of lead. The acid may be separated from the lead by the action of sulphuretted hydrogen; and then being filtered and evaporated, it will crystalize. Insoluble oxalates, the bases of which form insoluble compounds with sul′, may be decomposed by the action of this acid, when the ox′ will be separated.

*Action. Uses.* A virulent poison, which is very speedy in its action. Acute pain is immediately experienced, followed by vomiting. Great depression of the circulation ensues, nervous symptoms, such as great debility, numbness, etc., sometimes followed by convulsions. "But death follows so speedily after the injection of large doses, few of those who have died survived above an hour, that the symptoms have not been fully made out." Irritation and corrosion of the stomach are observed.

*Antidotes.* Chalk, whiting, or magnesia, mixed up with water, should be administered as quickly as possible in large quantities. Evacuate the stomach. Large quantities of water may be also useful."

